# Supplementary material for: Inference and Visualization of Complex Genotype–Phenotype Maps
Source: Mol Biol Evol. 2026 Feb 3;43(2):msag023. doi: 10.1093/molbev/msag023 (PMC12911936; doi:10.1093/molbev/msag023)
Supplement: msag023_Supplementary_Data [file msag023_supplementary_data.zip › supp_figures.pdf]

Supplementary Figures: Inference and visualization of complex genotype-phenotype maps

Carlos Martí-Gómez[1], Juannan Zhou[2,3], Wei-Chia Chen[4], Arlin Stoltzfus[5], Justin B. Kinney[1], and David M. McCandlish[1]

[1]Simons Center for Quantitative Biology, Cold Spring Harbor Laboratory, Cold Spring Harbor, NY, 11724, USA
[2]Department of Biology, University of Florida, Gainesville, FL, 32611, USA
[3]University of Florida Genetics Institute, University of Florida, Gainesville, FL, 32611, USA
[4]Department of Physics, National Chung Cheng University, Chiayi 62102, Taiwan, Republic of China
[5]Institute for Bioscience and Biotechnology Research, Rockville, MD, 20850, USA

February 6, 2026

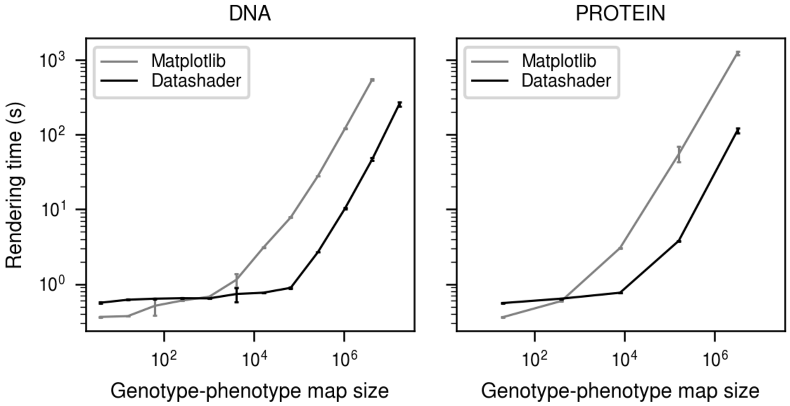

Figure S1: Visualization rendering times using two different back-end libraries for plotting as a function of the size of DNA and protein genotype-phenotype maps.

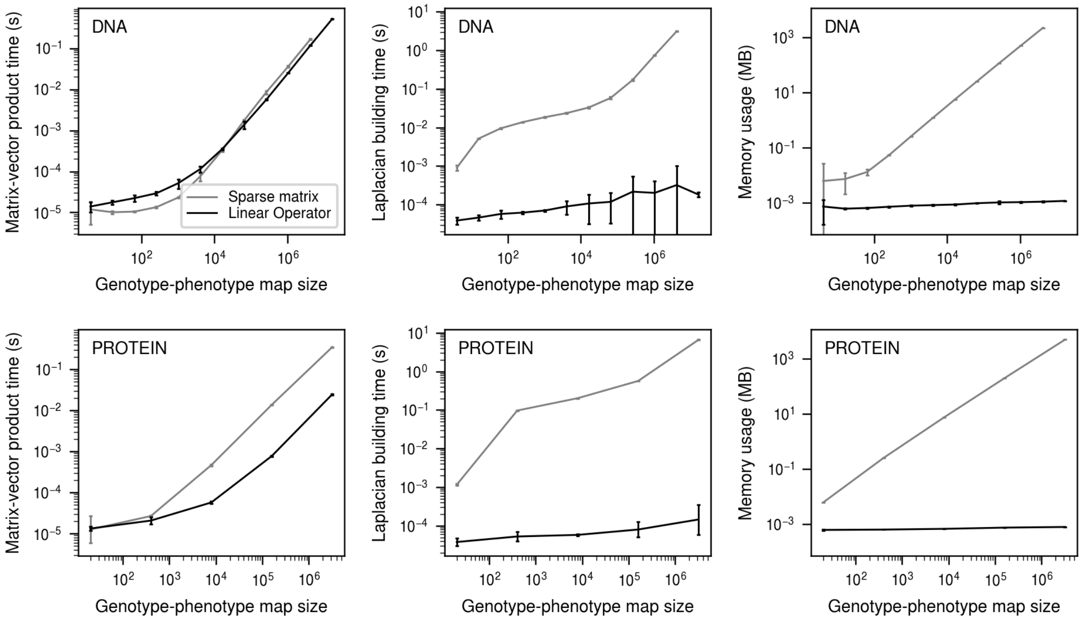

Figure S2: Comparison of the running times and memory requirements for computation of matrix-vector products with the Laplacian of the Hamming graph using our new Linear Operator or our previous sparse matrix formulation.

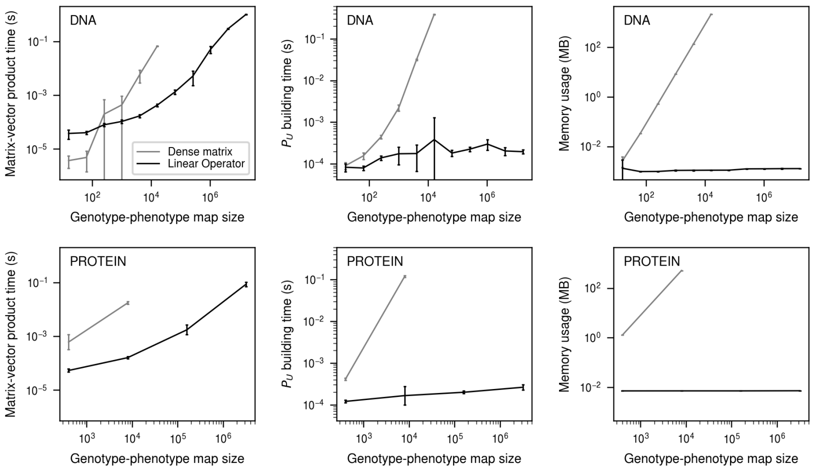

Figure S3: Comparison of the running times and memory requirements for computation of P_U matrix-vector products using our Linear Operator or the corresponding dense matrix.

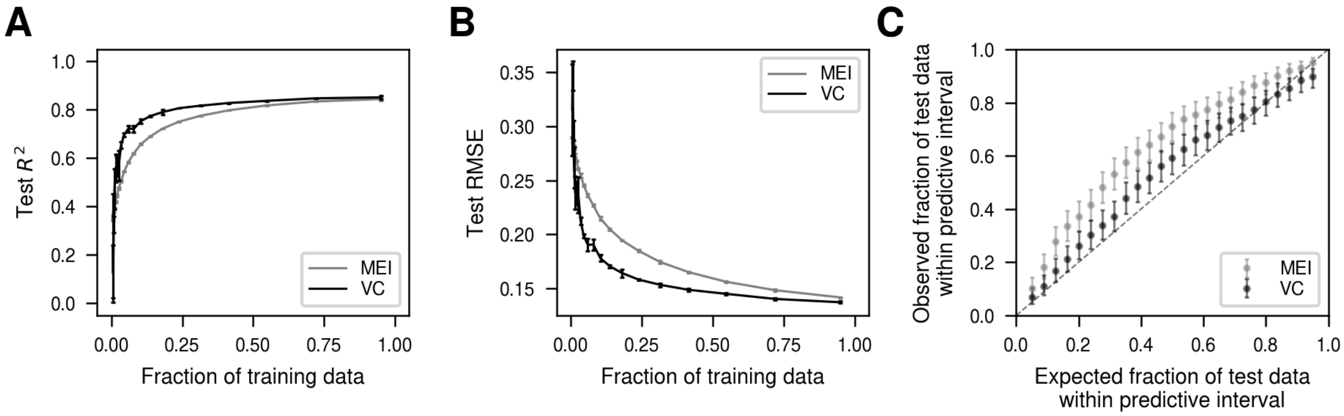

Figure S4: Predictive performance of Minimum Epistasis Interpolation (MEI) and Variance Component (VC) regression in held-out data. (A,B) Model predictive performance measured by the R^2 (A) and RMSE (B) in held-out data as a function of the fraction of data used for training and phenotypic prediction. Error bars represent the standard deviation over 3 independent subsets of sequences used for training at each proportion. (C) Evaluation of the models calibration by comparing the expected fraction of times a predictive interval will contain the real phenotypic value compared to the fraction of times it actually contained the measured phenotype across 274 test data points. Error bars represent the 95% Jeffreys confidence interval for the estimated fraction of data points laying within the corresponding predictive interval. Diagonal dashed gray line shows the expectation under perfect model calibration.

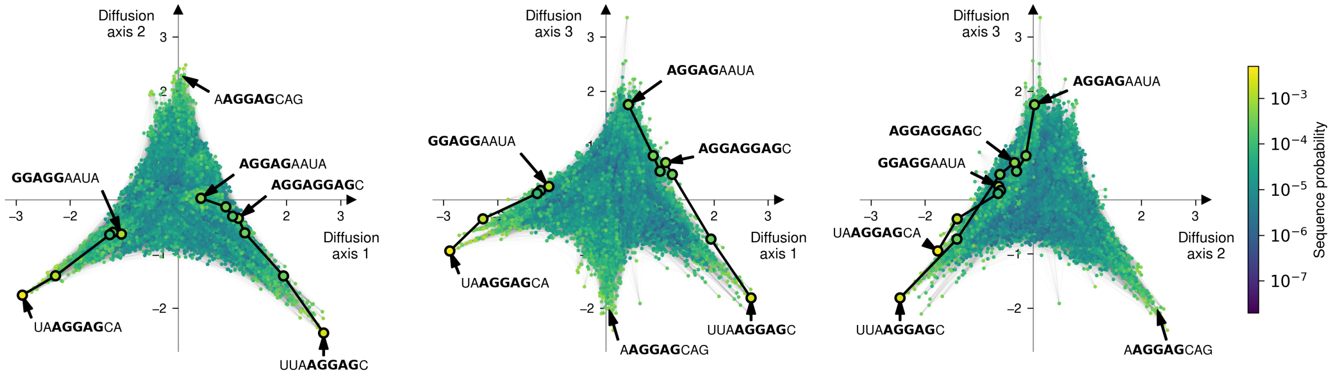

Figure S5: Low-dimensional representation of the Shine-Dalgarno probability distribution inferred with SeqDEFT along Diffusion axes 1, 2 and 3. Every dot represents one of the 4^9 possible sequences and is colored according to their inferred probability. Sequences are laid out according to the indicated Diffusion axes and dots are plotted in order according to the missing Diffusion axis.

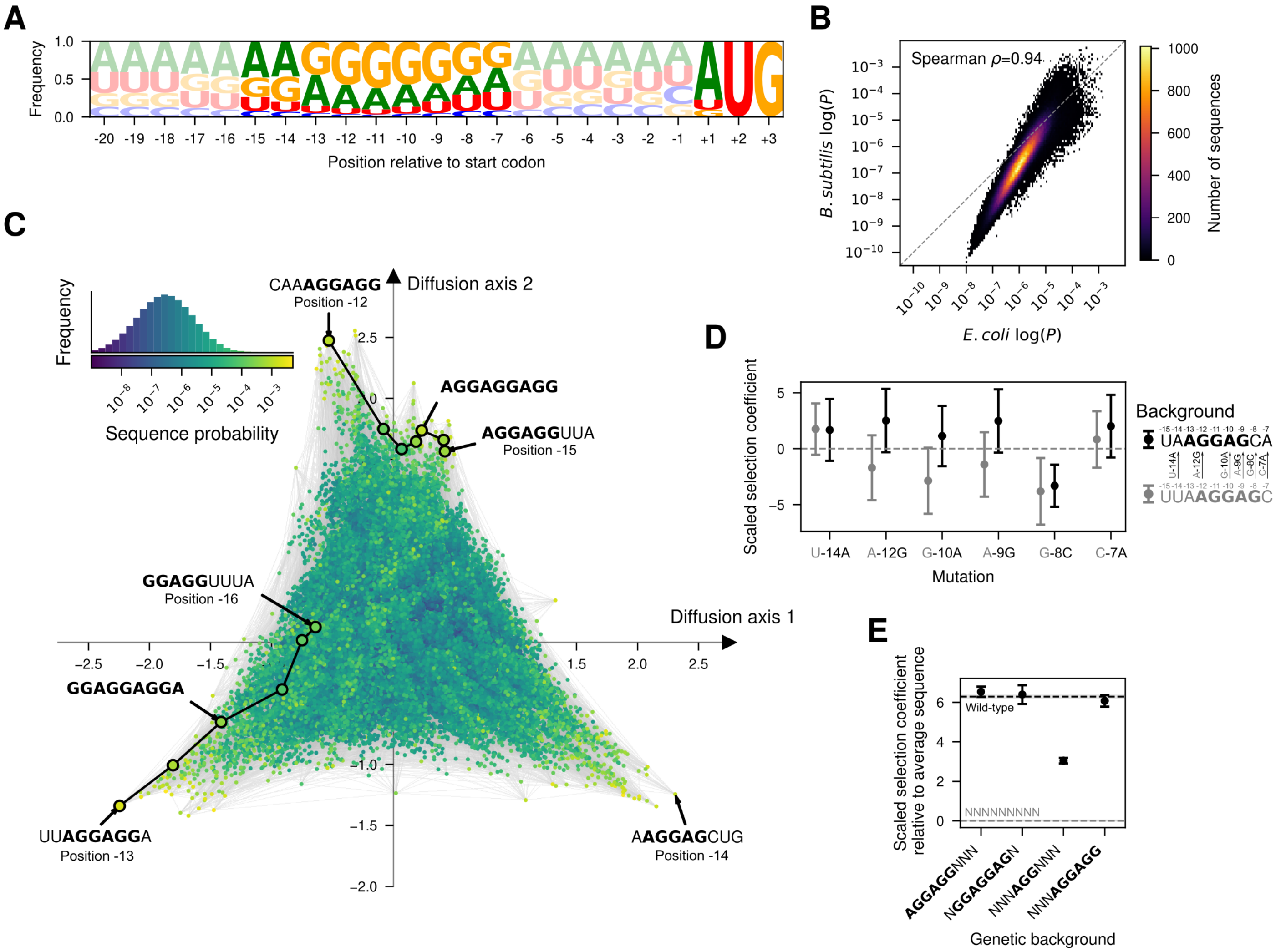

Figure S6: The structure of the genotype-phenotype map inferred from *B. subtilis* is conserved. (A) Sequence logo representing the site-specific allele frequencies of 4,328 5'UTRs in the *B. subtilis* genome aligned with respect to the annotated start codon. The start codon and the 9 nucleotide sequences 6 bases upstream are highlighted to emphasize the most relevant cis-regulatory sequences for translation initiation. (B) Two-dimensional histogram representing the relationship between the inferred sequence probabilities from their frequency in the *E. coli* and *B. subtilis* genomes. (C) Low-dimensional representation of the Shine-Dalgarno probability distribution inferred with SeqDEFT. Every dot represents one of the 4^9 possible sequences and is colored according to its inferred probability. The inset represents the distribution of inferred sequence probabilities along with their corresponding color in the visualization. Sequences are laid out according to the first two Diffusion axes and dots are plotted in order according to the 3rd Diffusion axis. (D) Posterior distribution inferred by SeqDEFT for the scaled selection coefficient of specific mutations when introduced in two genetic contexts, UUAAGGAGC and UAAGGAGCA, representing a shift of the AGGAG motif by one nucleotide. Note that estimated mutational effects, except for G-8C, are largely compatible with those estimated from the *E. coli* genome shown in Figure 4D in the UAAGGAGCA context. (E) Posterior distribution inferred by SeqDEFT for the average scaled selection coefficient, relative to the average across all possible sequences, for genotypes containing the AGGAGG motif at positions separated by three nucleotides, along with their potential mutational intermediates. Horizontal dashed lines represent posterior mean of the average phenotype across all possible sequences (grey) or wild-type genomic sequences (black). Shaded areas represent the 95% credible intervals. (D,E) Points represent the maximum a posteriori (MAP) estimates and error bars represent the 95% credible intervals.

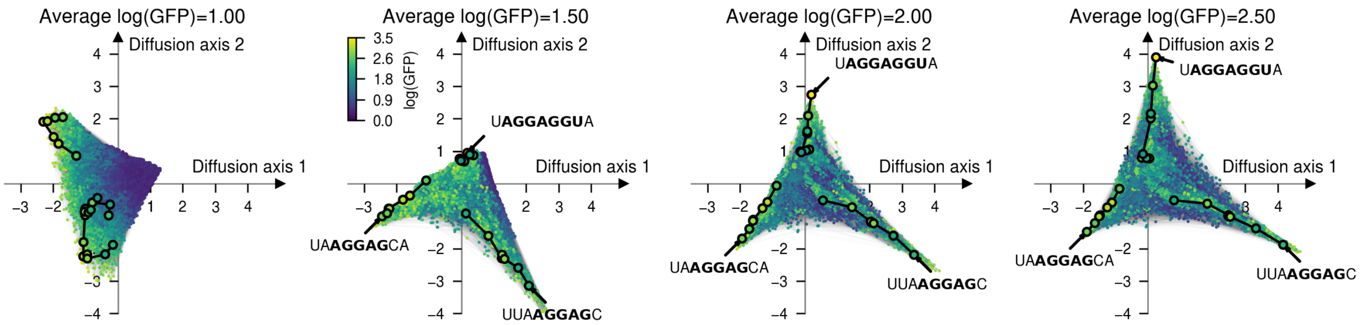

Figure S7: Low-dimensional representation of the Shine-Dalgarno genotype-phenotype map inferred with VC regression from MAVE data along Diffusion axes 1 and 2 as a function of the assumed average log(GFP) at the stationary distribution (as determined by tuning the strength of selection parameter c). Every dot represents one of the 4^9 possible sequences and is colored according to its inferred log(GFP) values. Dots are plotted in order according to Diffusion axes 3.

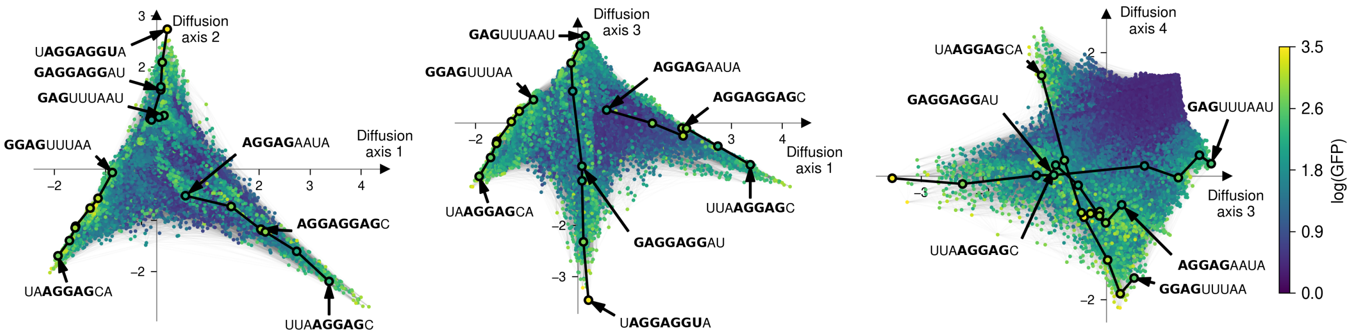

Figure S8: Low-dimensional representation of the Shine-Dalgarno genotype-phenotype map inferred with VC regression from MAVE data along Diffusion axes 1, 2, 3 and 4. Every dot represents one of the 4^9 possible sequences and is colored according to its inferred log(GFP) value. Sequences are laid out according to the indicated Diffusion axes and dots are plotted in order according to Diffusion axes 3, 2 and 1, respectively for each panel.

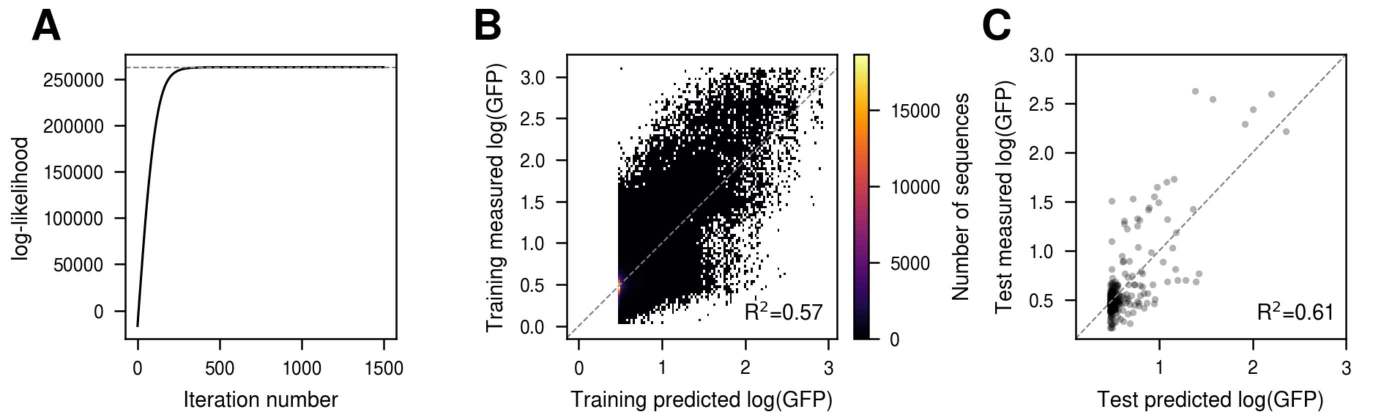

Figure S9: Fitting a thermodynamic model to the Shine-Dalgarno genotype-phenotype map using MAVE data. (A) Training curve showing the evolution of the log-likelihood as a function of the number of iterations of the Adam optimizer. (B) Comparison of measured log(GFP) in the training data with the predicted values under the estimated thermodynamic model. (C) Comparison of measured log(GFP) in the test data with the predicted values under the estimated thermodynamic model.

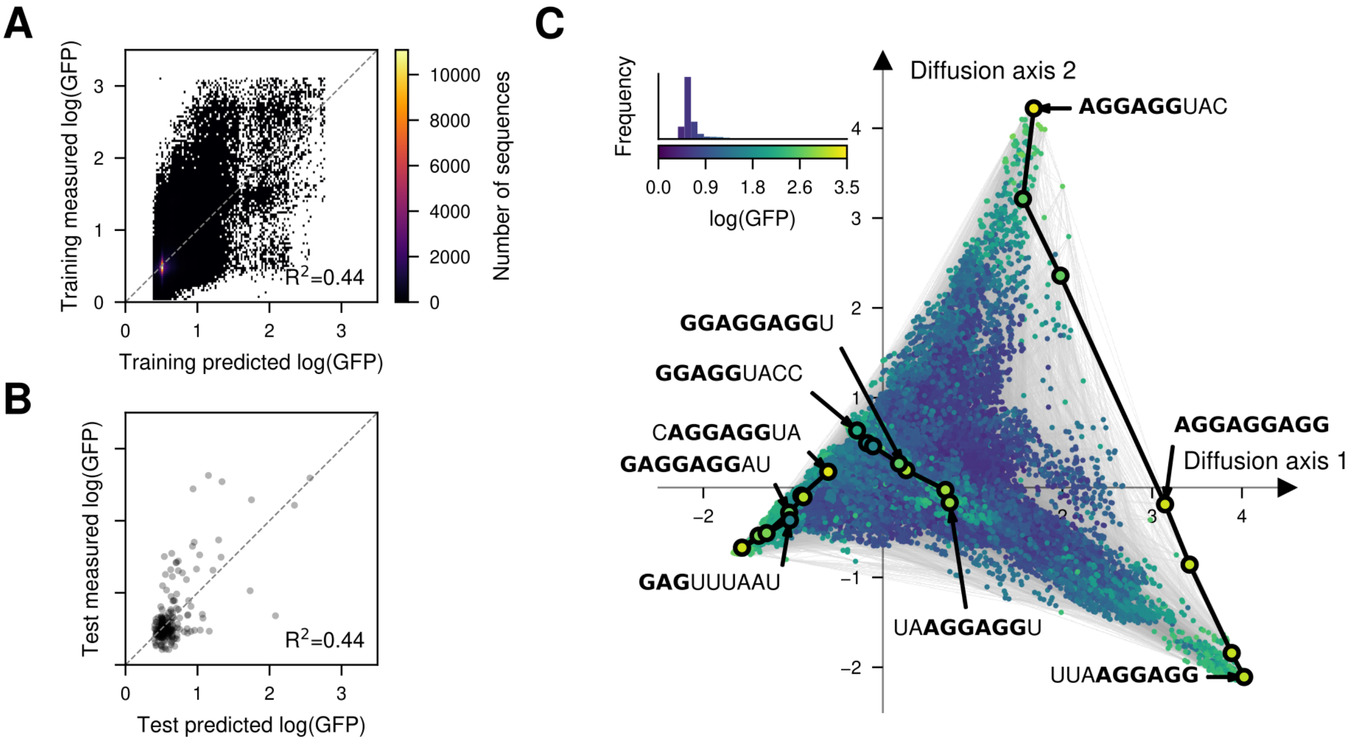

Figure S10: A general thermodynamic model for RNA folding for predicting MAVE data for the Shine-Dalgarno sequence. (A,B) Comparison of measured log(GFP) in the training (A) and test data (B) with the predicted values under the calibration model using RNAcofold ensemble binding energies with the anti-SD sequence. (C) Visualization of the genotype-phenotype map that results from predicting the phenotype of every possible sequence RNAcofold ensemble binding energies to the anti-SD sequence. Every dot represents one of the 4^9 possible sequences and is colored according to the predicted log(GFP). The inset represents the phenotypic distribution along with their corresponding color in the map. Sequences are laid out according to the first two Diffusion axes and dots are plotted in order according to Diffusion axis 3.
