# Supplementary material for: Inference and Visualization of Complex Genotype–Phenotype Maps
Source: Mol Biol Evol. 2026 Feb 3;43(2):msag023. doi: 10.1093/molbev/msag023 (PMC12911936; doi:10.1093/molbev/msag023)
Supplement: msag023_Supplementary_Data [file msag023_supplementary_data.zip › supp_info.pdf]

Supplementary Information: Inference and visualization of complex genotype-phenotype maps

Carlos Martí-Gómez[1], Juannan Zhou[2,3], Wei-Chia Chen[4], Arlin Stoltzfus[5], Justin B. Kinney[1], and David M. McCandlish[1]

[1]Simons Center for Quantitative Biology, Cold Spring Harbor Laboratory, Cold Spring Harbor, NY, 11724, USA
[2]Department of Biology, University of Florida, Gainesville, FL, 32611, USA
[3]University of Florida Genetics Institute, University of Florida, Gainesville, FL, 32611, USA
[4]Department of Physics, National Chung Cheng University, Chiayi 62102, Taiwan, Republic of China
[5]Institute for Bioscience and Biotechnology Research, Rockville, MD, 20850, USA

February 6, 2026

Contents

A Properties of the new projection operators **2**

B Variance explained by interactions among sets of sites **3**

C Linear operator for Kronecker products **4**

D Linear operator for Laplacian of the Hamming graph **5**

E Minimum epistasis interpolation solution and posterior distribution **5**

F Posterior distribution computation with the cost matrix **6**

A Properties of the new projection operators

Let f represent a genotype-phenotype map on the space of sequences with a single site and α different alleles. The function f can be projected into a subspace spanned by any vector b using the orthogonal projection matrix $P = b(b^T b)^{-1} b$. Letting V_{con} be the constant subspace spanned by the α-dimensional vector of ones $b = \vec{1}$, the orthogonal projection matrix into V_{con} is thus given by $P_{con} = \frac{1}{\alpha}\vec{1}\vec{1}^T$. Let V_{add} be the orthogonal subspace ($V_{con} \perp V_{add}$), defined by its projection matrix $P_{add} = I - P_{con} = I - \frac{1}{\alpha}\vec{1}\vec{1}^T$. Thus, for any pair of sequences x, y, $P_{con}(x, y) = \frac{1}{\alpha}$ and

$$P_{add}(x,y) = \begin{cases} \frac{-1}{\alpha} & \text{if } x \neq y \\ \frac{\alpha-1}{\alpha} & \text{if } x = y. \end{cases}$$

For a genotype-phenotype map f in the space of sequences of length ℓ, these elementary subspaces can be combined through tensor products into 2^ℓ different $V_U = \bigotimes_p^\ell V_p$ subspaces defined by the set of sites U, such that $V_p = V_{add}$ for $p \in U$ and $V_p = V_{con}$ for $p \notin U$. Thus, the projection operator into the subspaces defined by U are obtained through the Kronecker product $P_U = \bigotimes_p^\ell P_p$, the elements of which are

$$P_U(x,y) = \alpha^{-\ell} \prod_{\substack{p \in U \\ x_p \neq y_p}} (-1) \prod_{\substack{p \in U \\ x_p = y_p}} (\alpha - 1). \tag{1}$$

It is easy to show that the resulting subspaces V_U are orthogonal to each other using the mixed product property of the Kronecker product $(A \otimes B)(C \otimes D) = (AC) \otimes (BD)$. As a consequence, if either $AC = 0$ or $BD = 0$, then $A \otimes B$ and $C \otimes D$ are orthogonal. If we consider two subspaces defined by different subsets of sites U and U', there will be at least one position p at which the two site-specific elementary subspaces are orthogonal to each other, i.e., $P_p P_{p'} = 0$. Consequently, $P_U P_{U'} = (\bigotimes_p^\ell P_p)(\bigotimes_p^\ell P_{p'}) = \bigotimes_p^\ell (P_p P_{p'}) = 0$.

Next, we consider the subspaces defined by the direct sum of subspaces V_U defined by exactly k sites $V_k = \bigoplus_{U:|U|=k} V_U$ and derive the corresponding projection operator P_k:

$$\begin{aligned} P_k(x,y) &= \sum_{U:|U|=k} P_U(x,y) = \sum_{U:|U|=k} \alpha^{-\ell} \prod_{\substack{p \in U \\ x_p \neq y_p}} (-1) \prod_{\substack{p \in U \\ x_p = y_p}} (\alpha - 1) \\ &= \alpha^{-\ell} \sum_{U:|U|=k} \prod_{\substack{p \in U \\ x_p \neq y_p}} (-1) \prod_{\substack{p \in U \\ x_p = y_p}} (\alpha - 1). \end{aligned} \tag{2}$$

We note that the elements in the sum are obtained by multiplying the factors $(\alpha - 1)$ and (-1) together a total of k times. These products can take only $k+1$ possible values: $(-1)^q(\alpha-1)^{\ell-q}$ for $q = 0, 1, \ldots, \ell$, where q represents the number of missmatches between sequences x and y within sets of sites U. Therefore, P_k can be expressed by:

$$P_k(x,y) = \alpha^{-\ell} \sum_q^k (-1)^q (\alpha - 1)^{k-q} n_q(x,y), \tag{3}$$

where $n_q(x, y)$ is the number of times each unique value appears when summing through the corresponding P_U matrices. Because we are summing over all possible U of the same size, $n_q(x, y)$ does not depend on the specific sites or alleles at which x and y differ, but only on the Hamming distance between them $d(x, y)$. Specifically $n_q(x, y)$ is obtained by multiplying the number of ways to select q sites within the set of $d(x, y)$ different sites by the number of ways to select the remaining $k - q$ sites within the set of $\ell - d(x, y)$ matching sites:

$$P_k(x,y) = \alpha^{-\ell} \sum_{q}^{k} (-1)^q (\alpha-1)^{k-q} \binom{d(x,y)}{q} \binom{\ell - d(x,y)}{k-q} = \alpha^{-\ell} \mathcal{K}_k(d(x,y); \ell, \alpha), \tag{4}$$

where $\mathcal{K}_k(d(x,y); \ell, \alpha)$ denote the Krawtchouk polynomials (Stadler *et al.*, 1994; Zhou *et al.*, 2022). Note that this expression corresponds to the projection operator P_k into the subspace of pure k-th order interactions. Therefore, this shows that this k-th order interaction subspace V_k can be decomposed into smaller orthogonal subspaces V_U corresponding to pure k-th order interactions involving specific subsets of sites.

It is also easy to show that the columns of P_U are in the k-th eigenspace of the graph Laplacian by taking its projection and using the orthogonality properties of the P_U:

$$P_k P_U = \sum_{U':|U'|=k} P_{U'} P_U = \begin{cases} P_U & \text{if } |U| = k \\ 0 & \text{if } |U| \neq k. \end{cases} \tag{5}$$

Using the same argument, we can show that $P_U P_k = P_U \sum_{U':|U'|=k} P_{U'} = P_U$ when $|U| = k$ and 0 otherwise.

B Variance explained by interactions among sets of sites

In section A, we introduced the projection matrix P_U. This enables decomposing a genotype-phenotype map f into its 2^ℓ components f_U, each representing pure $|U|$-order interactions among sites in a set U. Here, we present two coarse-grained views of these components by quantifying the percentage variance explained by genetic interactions involving specific sites in a genotype-phenotype map f, where the total variance is given by

$$\text{Var}[f] = \alpha^{-\ell} (f - \bar{f})^T (f - \bar{f}), \tag{6}$$

where $\bar{f} = \alpha^{-\ell} \sum_i f_i$. In the first approach, we consider the variance explained by interactions of order k involving each site p in the sequence:

$$\text{Var}_k^p[f] = \alpha^{-\ell} (f_k^p)^T f_k^p, \tag{7}$$

where

$$f_k^p = \sum_{\substack{U:p \in U \\ |U|=k}} P_U f. \tag{8}$$

The values $100 \times \frac{\text{Var}_k^p[f]}{\text{Var}[f]}$ for every combination of k and p can be arranged in an $\ell \times \ell$-dimensional matrix to reveal which sites contribute most to variance, and how much through additive, low-order or higher-order genetic interactions.

In the second approach, we consider the variance explained by interactions of order $k = 2$ and $k > 2$ involving pairs of sites p, q:

$$\text{Var}_2^{p,q}[f] = \alpha^{-\ell} (f_2^{p,q})^T f_2^{p,q}, \tag{9}$$

$$\text{Var}_{k>2}^{p,q}[f] = \alpha^{-\ell} (f_{k>2}^{p,q})^T f_{k>2}^{p,q}, \tag{10}$$

$$f_2^{p,q} = \sum_{\substack{U:\{p,q\} \subseteq U \\ |U|=2}} P_U f, \tag{11}$$

$$f_{k>2}^{p,q} = \sum_{\substack{U:\{p,q\} \subseteq U \\ |U|>2}} P_U f, \tag{12}$$

These values can be summarized in an $\ell \times \ell$-dimensional matrix M

$$M_{p,q}(f) = \begin{cases} 100 \times \frac{\mathrm{Var}_2^{p,q}[f]}{\mathrm{Var}_2[f]} & \text{if } p < q \\ 0 & \text{if } p = q \\ 100 \times \frac{\mathrm{Var}_{k>2}^{p,q}[f]}{\sum_{k>2} \mathrm{Var}_k[f]} & \text{if } p > q, \end{cases} \tag{13}$$

where $\mathrm{Var}_k[f] = f_k^T f_k$. This matrix reveals the interaction patterns between pairs of sites, distinguishing pairwise from higher-order effects.

C Linear operator for Kronecker products

In section A, we describe the projection matrix P_U that projects a function f into the subspace corresponding to pure interactions between sites in a set U. Here we describe an efficient method for calculating the product $P_U f$ by noting that P_U can be written as a Kronecker product of ℓ matrices ($P_U = \bigotimes_p^\ell P_p$).

Let A be an arbitrary matrix obtained through an ℓ-Kronecker product, i.e., $A = \bigotimes_p^\ell A_p$ with entries given by $A(x, y) = \prod_p A_p(x_p, y_p)$ and $\mathbf{B} \in \mathbb{R}^{(\alpha \times \alpha \times \ldots \times \alpha)}$ be a tensor with ℓ dimensions such that $\mathbf{B}_{x_1, x_2, \ldots, x_\ell} = b_x$ for any vector $b \in \mathbb{R}^{\alpha^\ell}$. Thus, any matrix vector product Ab can be computed without explicitly constructing A using tensor dot products as follows:

$$\begin{aligned} (Ab)_x =& \sum_y A(x, y) b_y \\ =& \sum_{y_1, \ldots, y_\ell} \left(\prod_{p=1}^{\ell} A_p(x_p, y_p) \right) \mathbf{B}_{y_1, \ldots, y_\ell} \\ =& \sum_{y_2, \ldots, y_\ell} \left(\prod_{p=2}^{\ell} A_p(x_p, y_p) \right) \sum_{y_1} A_1(x_1, y_1) \mathbf{B}_{y_1, \ldots, y_\ell}. \end{aligned} \tag{14}$$

Let $\mathbf{B}^{(1)}_{x_1, y_2, \ldots, y_\ell} = \sum_{y_1} A_1(x_1, y_1) \mathbf{B}_{y_1, \ldots, y_\ell}$ and repeat the same operation:

$$\begin{aligned} (Ab)_x &= \sum_{y_2, \ldots, y_\ell} \left(\prod_{p=2}^{\ell} A_p(x_p, y_p) \right) \mathbf{B}^{(1)}_{x_1, \ldots, y_\ell} \\ &= \sum_{y_3, \ldots, y_\ell} \left(\prod_{p=3}^{\ell} A_p(x_p, y_p) \right) \sum_{y_2} A_2(x_2, y_2) \mathbf{B}^{(1)}_{x_1, y_2, \ldots, y_\ell} \\ &= \sum_{y_3, \ldots, y_\ell} \left(\prod_{p=3}^{\ell} A_p(x_p, y_p) \right) \mathbf{B}^{(2)}_{x_1, x_2, y_3, \ldots, y_\ell}. \end{aligned} \tag{15}$$

Thus, it is easy to see that $(Ab)_x$ corresponds to $\mathbf{B}^{(\ell)}_{x_1, \ldots, x_\ell}$ under the general recursion

$$\mathbf{B}^{(i)}_{x_1, \ldots, x_i, y_{i+1}, \ldots, x_\ell} = \sum_{y_i} A_i(x_i, y_i) \mathbf{B}^{(i-1)}_{x_1, \ldots, x_{i-1}, y_i, \ldots, y_\ell}, \tag{16}$$

where $\mathbf{B}^{(0)}_{y_1, \ldots, y_\ell} = \mathbf{B}_{y_1, \ldots, y_\ell} = b_y$. Note that each of the ℓ steps in the recursion can be computed efficiently as a tensor dot product between an $\alpha \times \alpha$ matrix and an $\alpha_1 \times_2 \alpha \times \cdots \times \alpha_\ell$ tensor requiring $\alpha^{\ell-1} \times \alpha^2$ operations.

The total number of required operations, $\ell\alpha^{\ell+1}$, is much smaller than the $\alpha^{2\ell}$ operations required for naively computing Ab, but more importantly, this strategy reduces memory requirements from the prohibitive scaling with $\alpha^{2\ell}$ for storing A to only α^{ℓ} for storing $\mathbf{B}$, enabling practical computation for α^{ℓ} in the order of millions.

D Linear operator for Laplacian of the Hamming graph

The space of possible sequences of length ℓ and α different alleles can be represented by a Hamming graph, in which nodes represent genotypes and edges represent single point mutations. The Laplacian matrix L of this graph is given by

$$L(i,j) = \begin{cases} -1 & \text{if } i \text{ and } j \text{ are neighbors,} \\ \ell(\alpha-1) & \text{if } i = j, \\ 0 & \text{otherwise.} \end{cases} \tag{17}$$

This matrix is sparse and can be stored in Compressed Sparse Row (CSR) format to efficiently compute matrix vector products. Despite the sparsity, there are still $\alpha^{\ell} \times (1 + \ell(\alpha - 1))$ non-zero entries. For the space of sequences of length 9 with 4 alleles with 64 bits floating point values, only storing the non-zero entries would require about 450MB. Here, we develop a matrix-free function to compute matrix-vector products with the Laplacian matrix Lb by leveraging the highly regular structure of this matrix and tensor broadcasting, resulting in memory requirements that scale only with the size of sequence space α^{ℓ}. In particular, we can express Lb as

$$Lb = (\ell(\alpha-1)I - A)b = (\ell\alpha I - (\ell I + A))b = \ell\alpha b - (\ell I + A)b = \ell\alpha b - w,$$

where A is the adjacency matrix and w is the product $(\ell I + A)b$. For a fixed choice of b, let $\mathbf{B} \in \mathbb{R}^{(\alpha\times\alpha\times\ldots\times\alpha)}$ be a tensor with ℓ dimensions such that $\mathbf{B}_{x_1,x_2,\ldots,x_\ell} = b_x$, where x represents a sequence and x_i the allele at position i. Then for the same choice of b, the tensor $\mathbf{W}$ having elements $\mathbf{W}_{x_1,x_2,\ldots,x_\ell} = w_x$ can be easily computed in tensor form using broadcasting by using the trick

$$\mathbf{W} = \sum_{i}^{\ell} \mathbf{B}^{(i)}. \tag{18}$$

$\mathbf{B}^{(i)}_{x_1,\ldots,x_{i-1},*,x_{i+1},\ldots,x_\ell} = \sum_c^{\alpha} \mathbf{B}_{x_1,\ldots,x_{i-1},c,x_{i+1},\ldots,x_\ell}$, where the '*' character indicates broadcasting, i.e. all characters at position i lead to the same value. Thus, this can be efficiently computed by summing the entries of tensor $\mathbf{B}$ over axis i. We can then use $\mathbf{B}$ and $\mathbf{W}$ to calculate $w = (\ell I + A)b$ as:

$$\begin{aligned} w_x = \mathbf{W}_{x_1,x_2,\ldots,x_\ell} &= \sum_{i}^{\ell} \sum_{c}^{\alpha} \mathbf{B}_{x_1,\ldots,x_{i-1},c,x_{i+1},\ldots,x_\ell} \\ &= \ell \mathbf{B}_{x_1,x_2,\ldots,x_\ell} + \sum_{i}^{\ell} \sum_{c \neq x_i}^{\alpha} \mathbf{B}_{x_1,\ldots,x_{i-1},c,x_{i+1},\ldots,x_\ell} \\ &= \ell b_x + (Ab)_x = ((\ell I + A)b)_x. \end{aligned}$$

E Minimum epistasis interpolation solution and posterior distribution

In this section, we derive the minimum epistasis interpolation solution as the maximum a posteriori (MAP) estimate of a Gaussian process model under a prior distribution on local epistatic coefficients. Let us consider a

complete genotype-phenotype map given by an α^ℓ-dimensional vector f and define an improper prior distribution defined by the precision matrix $C = \frac{a}{s}\Delta^P$, such that $\log \mathrm{p}(f) \propto -f^T C f$.

Assuming we know exactly the phenotypes f_x for a subset of sequences x, we aim to compute the posterior probability of the phenotypes f_z at all remaining unobserved sequences z given by $\mathrm{p}(f_z|f_x)$. Let us define the joint log-probability distribution over $[f_x, f_z]$ given by

$$\begin{aligned}\log \mathrm{p}(f) = \log p\left(\begin{bmatrix} f_x \\ f_z \end{bmatrix}\right) &\propto -\frac{1}{2} f^T C f = -\frac{1}{2}\begin{bmatrix} f_x^T & f_z^T \end{bmatrix}\begin{bmatrix} C_{xx} & C_{xz} \\ C_{xz} & C_{zz} \end{bmatrix}\begin{bmatrix} f_x \\ f_z \end{bmatrix} \\ &= -\frac{1}{2}\left(f_x^T C_{xx} f_x + 2 f_x^T C_{xz} f_z + f_z^T C_{zz} f_z\right).\end{aligned}$$

Since C_{zz} is a principal submatrix of the positive semidefinite matrix C, it is also positive semidefinite for any z, and thus $\log \mathrm{p}(f_z|f_x)$ is convex. We can now take the gradient $\nabla_{f_z} \log \mathrm{p}(f_z|f_x) = f_x^T C_{xz} f_z + C_{zz} f_z$ and find the MAP $\hat{f}_z$ as the solution to the equation $C_{zz}\hat{f}_z + C_{zx} f_x = 0$, which is unique if and only if the corresponding $P-1$-th order model is uniquely determined (Zhou and McCandlish, 2020) and is given by $\hat{f}_z = -C_{zz}^{-1} C_{zx} f_x$. In this case, it is easy to verify that the posterior covariance is given by C_{zz}^{-1} as

$$\begin{aligned}\log \mathrm{p}(f_z|f_x) &\propto (f_z + C_{zz}^{-1} C_{zx} f_x)^T C_{zz} (f_z + C_{zz}^{-1} C_{zx} f_x) = \\ &= f_z^T C_{zz} f_z + 2 f_x^T C_{xz} C_{zz}^{-1} C_{zz} f_z + f_x^T C_{xz} C_{zz}^{-1} C_{zz} C_{zz}^{-1} C_{zx} f_x \\ &= f_z^T C_{zz} f_z + 2 f_x^T C_{xz} f_z + f_x^T C_{xz} C_{zz}^{-1} C_{zx} f_x \propto f_z^T C_{zz} f_z + 2 f_x^T C_{xz} f_z.\end{aligned} \tag{19}$$

In the case in which $C = \frac{a}{s}\Delta^{(P)}$, the posterior mean $\hat{f}_z = -(\Delta_{zz}^{(P)})^{-1}\Delta_{zx}^{(P)} f_x$ is independent of a. The posterior covariance, on the other hand, is independent of f_x, but depends on a and the pattern of observations. Thus, if we want to compute the variance of the phenotypic predictions of specific sequences, we define the a^* such that the expected sum of squared local epistatic coefficients $\mathbb{E}[\epsilon_P^2] = \frac{1}{a}\mathrm{rank}(\Delta^{(P)})$ under the prior (Chen *et al.*, 2021) matches the one under the posterior mean given by $\frac{1}{s_P}\hat{f}^T \Delta^{(P)} \hat{f}$.

F Posterior distribution computation with the cost matrix

Given a set of n measurements y in a subset of sequences x with measurement variances arranged along the diagonal of an $n \times n$ matrix D, we aim to obtain the complete genotype-phenotype map represented by the α^ℓ-dimensional vector $\hat{f}$ that maximizes the posterior probability of f given the observations y, i.e., we wish to find

$$\hat{f} = \arg\max_f \log \mathrm{p}(f|y).$$

We begin by defining an $\alpha^\ell \times n$ matrix X relating the points in the complete space with the n observed values, such that $f_x = X^T f$

$$X_{ij} = \begin{cases} 1 & \text{if observation } j \text{ corresponds to sequence } i \\ 0 & \text{otherwise.} \end{cases}$$

Using X, we can then write the posterior log-probability as a function of f:

$$\log \mathrm{p}(f|y) \propto -\frac{1}{2} f^T C f - \frac{1}{2}(y - X^T f)^T D^{-1} (y - X^T f). \tag{20}$$

We can then expand this expression to separate factors that depend on f from those that depend only on the data y.

$$\log \mathrm{p}(f|y) \propto -\frac{1}{2}f^T C f - \frac{1}{2}y^T D^{-1} y + f^T X D^{-1} y - \frac{1}{2} f^T X D^{-1} X^T f$$
$$= -\frac{1}{2} f^T (C + X D^{-1} X^T) f + f^T X D^{-1} y - \frac{1}{2} y^T D^{-1} y.$$

We next take the gradient with respect to f

$$\nabla_f \log \mathrm{p}(f|y) = -(C + XD^{-1}X^T)f + XD^{-1}y,$$

and solve for $\hat{f}$ by setting $\nabla_f \log \mathrm{p}(f|y) = 0$, which yields

$$\hat{f} = (C + XD^{-1}X^T)^{-1} X D^{-1} y. \tag{21}$$

If C is invertible, then we can define a kernel matrix $K = C^{-1}$ over the complete genotype-phenotype map and verify that this is equivalent to the classical solution for the posterior mean of a Gaussian process model using Woodbury's identity. Specifically, for a subset of sequences z and the α^ℓ by $|z|$ matrix Z defined by:

$$Z_{ij} = \begin{cases} 1 & \text{if sequence } i \text{ is the } j\text{-th member of } z \\ 0 & \text{otherwise,} \end{cases}$$

we find that

$$\begin{aligned}
\hat{f}_z &= Z^T \hat{f} \\
&= Z^T (C + XD^{-1}X^T)^{-1} X D^{-1} y \\
&= Z^T \left(K - KX(X^T K X + D)^{-1} X^T K\right) X D^{-1} y \\
&= Z^T K \left(I - X(X^T K X + D)^{-1} X^T K\right) X D^{-1} y \\
&= Z^T K \left(X - X(X^T K X + D)^{-1} X^T K X\right) D^{-1} y \\
&= Z^T K X \left(I - (X^T K X + D)^{-1} X^T K X\right) D^{-1} y.
\end{aligned}$$

Then we can use the fact that $I = (X^T K X + D)^{-1}(X^T K X + D)$ to obtain the identity:

$$\begin{aligned}
& I - (X^T K X + D)^{-1} X^T K X \\
&= (X^T K X + D)^{-1}(X^T K X + D) - (X^T K X + D)^{-1} X^T K X \\
&= (X^T K X + D)^{-1}(X^T K X + D - X^T K X) \\
&= (X^T K X + D)^{-1} D.
\end{aligned}$$

Substituting this identity into our previous expression for $\hat{f}_z$, we now recover the classical maximum a posteriori solution for Gaussian process regression

$$\hat{f}_z = Z^T K X \left(X^T K X + D\right)^{-1} D D^{-1} y = Z^T K X \left(X^T K X + D\right)^{-1} y,$$

as desired.

Turning to the covariance matrix for the posterior, knowing that the posterior distribution is multivariate Gaussian implies that the posterior covariance matrix is given by the inverse of the Hessian matrix of the log-posterior probability. Thus the covariance matrix of the posterior distribution is given by:

$$\Sigma = (\nabla\nabla_f log(f|y))^{-1} = \left(C + XD^{-1}X^T\right)^{-1}, \tag{22}$$

which we note depends on our observations only through the pattern of observed sequence as encoded in X and not on the observed phenotypes y.

Based on the marginalization property of multivariate Gaussian distributions, the posterior covariance at a subset of points z can be obtained simply by taking the submatrix $\Sigma_{zz} = Z^T \Sigma Z$. We can verify that this also matches the classical solution for Gaussian process posterior covariance when $K = C^{-1}$ using Woodbury's identity:

$$\begin{aligned}
\Sigma_{zz} &= Z^T \left(C + XD^{-1}X^T\right)^{-1} Z \\
&= Z^T \left(C^{-1} - C^{-1}\left(X^T C^{-1} X + D\right)^{-1} C^{-1}\right) Z \\
&= Z^T \left(K - K\left(X^T K X + D\right)^{-1} K\right) Z \\
&= Z^T K Z - Z^T K \left(X^T K X + D\right)^{-1} K Z \\
&= K_{zz} - K_{zx}\left(K_{xx} + D\right)^{-1} K_{xz},
\end{aligned} \tag{23}$$

as desired.

References

Chen, W.-C., Zhou, J., Sheltzer, J. M., Kinney, J. B., and Mccandlish, D. M. 2021. Field-theoretic density estimation for biological sequence space with applications to 5 splice site diversity and aneuploidy in cancer. *Proc. Natl. Acad. Sci. USA*.

Stadler, P. F., Happel, R., *et al.* 1994. Canonical approximation of landscapes. *Santa Fe Institute Preprint*, pages 94–09.

Zhou, J. and McCandlish, D. M. 2020. Minimum epistasis interpolation for sequence-function relationships. *Nature Communications*, 11(1).

Zhou, J., Wong, M. S., Chen, W.-c., Krainer, A. R., Justin, B., and Mccandlish, D. M. 2022. Higher-order epistasis and phenotypic prediction. *Proc. Natl. Acad. Sci. USA*, 119(39).
